# Induced Pluripotent Stem Cell Modeling of Gaucher’s Disease: What Have We Learned?

**DOI:** 10.3390/ijms18040888

**Published:** 2017-04-21

**Authors:** Dino Matias Santos, Gustavo Tiscornia

**Affiliations:** 1 Department of Biomedical Sciences and Medicine, University of Algarve, Faro 8005-139, Portugal; dinomatias@gmail.com; 2 Center for Biomedical Research, University of Algarve, Faro 8005-139, Portugal; 3 Clínica EUGIN, Barcelona 08028, Spain

**Keywords:** lysosomal storage disease, Gaucher’s disease, glucocerebrosidase, induced pluripotent stem cells, chaperone therapy

## Abstract

Gaucher’s disease (GD) is the most frequently inherited lysosomal storage disease, presenting both visceral and neurologic symptoms. Mutations in acid β-glucocerebrosidase disrupt the sphingolipid catabolic pathway promoting glucosylceramide (GlcCer) accumulation in lysosomes. Current treatment options are enzyme replacement therapy (ERT) and substrate reduction therapy (SRT). However, neither of these approaches is effective in treating the neurological aspect of the disease. The use of small pharmacological compounds that act as molecular chaperones is a promising approach that is still experimental. In recent years, an association between GD and Parkinson like synucleinopathies has been discovered. Since 1992, a number of mouse models of GD have been the developed and partially reproduce phenotype of the disease. More recently, the discovery of direct reprograming has allowed the derivation of induced pluripotent stem cells (iPSc) from fibroblasts obtained from GD patients. iPSc can be expanded indefinitely in vitro and differentiated to macrophages and neurons, the main relevant cell types involved in GD. In this work, we review iPSc models of GD and summarize what we have learned from this system.

## 1. Introduction

Gaucher’s disease (GD) is the most common autosomal, recessively inherited lysosomal storage disease (LSD). The disorder is highly variable in symptoms, severity and age of onset. The symptoms affect multiple organs and systems, and can be broadly divided into systemic (or visceral) and neurological symptoms. The systemic symptoms include hepato and splenomegaly, anemia, pancytopenia, osteopenia, osteoporosis and bone pain due to infarction or fractures. The neurological symptoms include spasticity, seizures, eye movement impairment, cognitive problems and neurodegeneration [1,2].

Severity and age of onset are inversely correlated and may vary from asymptomatic individuals and patients mildly affected very late in life, to congenital manifestations of extreme severity. GD is routinely classified as non-neuronopathic (type 1), acute neuronopathic (type 2) and chronic neuronopathic (type 3). The different GD types vary in symptoms, organ systems affected, severity, age of onset, incidence and prevalence in different ethnic groups (Table 1). However, it has become increasingly recognized that patients can be positioned on a phenotypic continuum spanning from mild oculomotor abnormalities to perinatal death, rather than neatly fall into a specific category of disease type [3]. GD type 1 accounts for over 90% of all cases and is relatively milder that GD type 3 or type 2, presenting only visceral symptoms. Both GD type 3 and type 2 show exacerbated visceral symptoms along with neurological symptoms. Type 2 (acute neuronopathic) is the most severe form of the disease developing severe neurological symptoms within the first six months of life; patients rarely survive their third birthday. Type 3 GD (chronic neuronopathic) represents an intermediate form of the disease, presenting later onset than GD type 2 and showing both visceral and neurological symptoms [4].

The disease is caused by mutations in the gene encoding for the lysosomal enzyme acid β-glucocerebrosidase, which hydrolyzes glucosylceramide to glucose and ceramide within the context of a general catabolic pathway of complex sphingolipids [5,6,7]. GCase is broadly expressed across cell types and formed by three distinct domains; the catalytic site is located in Domain 3 [8]. There have been over 300 identified mutations in *GBA1* known to result in some type of GD phenotype, including point mutations, splice-site mutations, deletions, insertions, complex alleles (with more than one mutation) and recombinant alleles containing genomic sequences of both the *GBA1* gene and a highly homologous pseudogene located 16 kb downstream [9]. However, only four mutations (N370S, L444P, 84GG and IVS2+1) account for 90% of GD cases in Ashkenazi Jews and 49% in non-Jewish populations [4]. Mutations do not cluster to the catalytic site; rather, they are distributed relatively evenly throughout the sequence and lead to a miss-folding of the enzyme that results in miss-trafficking to the lysosome and markedly lowered enzymatic activity [10,11,12,13,14]. GD patients with miss-folding mutations typically have 5–20% enzymatic activity; however, some mutations result in no protein production [15]. In contrast, heterozygotes have approximately 50% activity and generally lack overt symptoms. The genotype-phenotype correlation is weak and it is not possible to accurately predict the clinical presentation caused be a particular mutation, suggesting that multiple factors which might be involved in GD pathogenesis [3]. However, the N370S mutation (in homozygosis or as a compound heterozygote) correlates with GD type 1, while the L444P mutation is associated with neuronopathic GD. The L444P homozygous genotype tends to produce GD type3, while L444P in heterozygosis with a recombinant allele is associated with GD type 2 [16,17]. GCase is synthesized on polyribosomes and immediately translocated into the ER where it is post-translationally modified; its signal peptide is excised and *N*-linked glycans (mainly mannose-6P) are added on four asparagine residues (N19, N59, N146, and N270); only N19 glycosylation is required for catalytic activity [18]. The presence of conformational alterations often caused by mutations in *GBA1* is recognized by the endoplasmic reticulum (ER) quality control machinery. Miss-folded proteins are ubiquitinated by a E3 Ubiquitin ligase on residue K48, subsequently triggering either ER Associated Degradation (ERAD) or the ubiquitin–proteasome (UPS) degradation pathways [19,20,21]. If GCase passes ER quality control system, it is then trafficked to the lysosome mainly via a lysosomal membrane protein 2 (LIMP-2) mediated process [22]. Lowered levels of GCase activity in the lysosome block the sphingolipid degradative pathway and lead to GlcCer accumulation within lysosomes of cells. Neurons and macrophages are particularly affected, with macrophages originating the characteristic lipid laden cells (Gaucher cells), which constitute the histological hallmark of the disease. Macrophages migrating into different organs explain the multisystem clinical presentation of the disease. However, the effects of the mutated GCase protein on cellular physiology and pathways are many, and the mechanistic details underlying the pathology subject of intensive research.

## 2. Therapeutic Approaches

A number of approaches to ameliorate symptoms or cure the disease have been explored experimentally and/or clinically, including enzyme replacement therapy (ERT), substrate reduction therapy (SRT), chaperone therapy, bone marrow transplant and gene therapy. ERT involves periodic infusions of wild type (WT) GCase recombinant protein (imiglucerase, velaglucerase alfa, or taliglucerase alfa) into the bloodstream, where the recombinant enzyme is taken up by macrophages through mannose receptor mediated endocytosis, eventually reaching the lysosome. ERT is the treatment of choice for the systemic symptoms of GD, resulting in decreased hepato and splenomegaly and a notable increase in patient quality of life; other organs, such as lungs and bones, respond less well to treatment. However, due to its high molecular weight, the recombinant enzyme cannot cross the blood–brain barrier and is therefore not effective in treating neuronopathic symptoms [23]. Disadvantages of ERT are its high cost and the need for regular infusions [24]. SRT uses a pharmacological inhibitor (*N*-butyl deoxynojirimycin) of glucosyl-ceramide synthase, the enzyme that catalyzes the synthesis of glucosylceramide. In decreasing the rate of synthesis, SRT results in a net decrease in the levels of glucosylceramide. While these compounds are small enough to cross the blood–brain barrier, no improvement in neurological symptoms has been reported to date in a clinical setting [23]. Some of the most frequent mutations affect enzyme stability and cause a decrease in trafficking to the lysosome, rather than affecting the active site and enzyme catalytic activity. This has led to the development of a therapeutic strategy based on pharmacological compounds (chaperones) capable of interacting with the enzyme in such a way that its three-dimensional structure is stabilized. As a result, the correctly folded GCase can escape the ER control system and be correctly trafficked though the secretory pathway to reach the lysosome. The small size of these compounds allows them to cross the blood–brain barrier, making them promising candidates for ameliorating or preventing neuronopathic symptoms of the disease. A number of compounds with different chemistries have been developed [24]. Most of these molecules target the active site and must therefore be reversible inhibitors, ideally with shorter half-lives that the substrate, but compounds that interact with other regions of the protein could in principle also work. Recently, pH sensitive chaperones have been developed which bind strongly to the GCase active site at neutral pH but lose affinity when exposed to the lower pH found in the lysosome [25]. While some chaperones have reached human clinical trials, to date none has shown any significant improvement in clinical outcome. Bone marrow transplant has been used for a small number of patients with GD with acceptable results [26]; however, due to the relatively high risk of this approach and the availability of ERT and SRT, this therapeutic option has mostly been abandoned. Gene therapy approaches remain experimental [26,27].

## 3. Relation with Parkinson’s Disease

Parkinson’s disease (PD) is the second most common neurodegenerative disease (after Alzheimer’s disease) affecting over 1% and 4% of people over 65 and 85 years of age, respectively [28,29]. It is characterized by tremor, postural instability, rigidity and bradykinesia as well as non-motor symptoms such as cognitive deficiencies, psychosis, sleep alterations and depression [29]. Its histological hallmark is accumulation of insoluble aggregates of α-synuclein in neurons and glia of the substantia nigra [30]. The synuclein family consists of four members (α-synuclein, β-synuclein, γ-synuclein and synoretin) with sizes between 127 and 140 aa whose functions are poorly known; α-synuclein is thought to be involved in synaptic function [31]. Both polygenic inheritance and environmental factors are thought to cause the disease [32]. Eighteen loci involved in PD have been identified though gene wide association and genetic linkage studies [32,33,34,35]. Over the last 15 years, a number of lines of evidence have revealed an unexpected link between GD and the synucleinopathies. Initial case reports, some as far back as 1985, described GD patients with Parkinson’s like symptoms [36,37,38]. Moreover, post-mortem examination of brains from patients with GD type 1 and PD showed similar pathophysiologies characterized by α-synuclein accumulation, presence of Lewy bodies in the substantia nigra and hippocampal pyramidal cell layers, and loss of pigmented neurons [39,40]. Analysis of family trees of GD patients showed a larger than expected frequency of relatives with PD, many of them *GBA1* heterozygotes [41,42]. Furthermore, several independent analyses of PD patient cohorts revealed increased frequency of *GBA1* mutations [43,44,45,46,47]. However, these studies suffered from low patient numbers or focused on a subset of GD mutations. In 2009, a milestone study involving a multicenter study (16 centers in 12 countries) compared the full length *GBA1* sequence in 5691 PD patients to that of 4898 healthy controls, uncovering significant associations between both diseases [48]. *GBA1* mutations were found in 6.9% of PD patients but only in 1.3% of the controls. In the Ashkenazi Jewish subset, 19.3% of PD patients had *GBA1* mutations, compared to only 4.1% in the controls. In addition, *GBA1* mutation carriers trended towards developing Parkinsonian symptoms four years earlier than non-carriers and had a greater probability of having a history of PD in the family. Interestingly, *GBA1* mutation carriers tended to have less bradykinesia and rest tremor but higher levels of cognitive impairments than controls. No association with any particular *GBA1* mutation was found [48]. Similar studies have corroborated these findings and mutations in *GBA1* are currently considered the main risk factor for developing PD [49,50,51]; individuals with homozygous or heterozygote *GBA1* mutations have a 20–30-fold increased chance of developing PD [48,49,50].

## 4. Experimental Models of Gaucher’s Disease (GD)

The main avenues for studying the mechanisms of GD disease are patient tissue specimens, in vitro culture models and animal models.

Patient tissue specimens allow direct study of disease pathophysiology. Human primary cultures can be established from cell types that are both available and amenable to in vitro culture, such as monocytes or dermal fibroblasts. Of these two, only monocytes are a primary cell type involved in the disease. Monocytes can be isolated from blood and differentiated into macrophages for study, but their expansion capacity is generally low unless key transcription factors such as MafB are downregulated [52]. Fibroblast can be obtained from skin biopsies and can be expanded for a limited number of passages, generating enough cells for research; however, fibroblasts are not a cell type primarily involved in the disease. Primary cultures can be immortalized, but doing so may result in loss of the normal physiological conditions present in GD patients, limiting the types of experiments that can be undertaken. Obtaining material, the main tissues affected in the disease from visceral organs or the CNS of live patients is understandably impractical. Several studies have analyzed post-mortem tissues (particularly brain) [40]; however, in these cases researchers are limited to analyzing advanced stages of the disease [53,54,55].

A number of mouse models have been developed for GD. The first approach to generate a GD mouse model focused in GCase inhibition with the conduritol-β-epoxide (CBE). Daily CBE intraperitoneal injections during the course of three weeks decreased GCase´s activity up to 90% in mice [56,57,58]. After treatment, the levels of GlcCer were higher in spleen, liver and brain (up to five-fold) [56]. Five-day-old mice subjected to 10 days of daily CBE injections developed severe neuronopathic symptoms myoclonus, tremor and incoordination and limb dragging and died [58]. Interestingly, age matched animals subjected for the same treatment for 5–6 days suffered a period of latency of 3–4 days before showing progressive and persistent neurological symptoms starting with myoclonus, tremor and incoordination and limb dragging, suggesting that once started, the neurodegenerative process might not be reversible even if GCase levels are reestablished [58].

Further increase in GlcCer levels has been achieved by injecting a mixture of CBE with GlcCer. Liposomal administration of GlcCer, after depletion of the endogenous GCase levels with CBE, led to over 90% reduction in GCase activity levels. Furthermore, the intracellular levels of GlcCer in Kupffer cells increased 5–10 fold for as long as five days [59,60]. This enhanced chemical model of visceral GD has proven very useful to demonstrate the effectiveness of in vivo gene therapy using low doses of a retroviral vector expressing GCase. According to Marshall et al., systemic delivery of 5×10^12^ particles/kg of GCase expressing adenoviral particles was enough to generate therapeutic GCase levels in the serum [60].

In 1992 the first genetic model of GD was created by inserting a Neo cassette in the presumptive GCase active site, exons 9–10, by homologous recombination. Mice carrying the disrupted gene produce an inactive form of the enzyme, resulting in a null *GBA1* allele (nGBA). Homozygous mice for nGBA showed <4% GCase activity and higher levels of GlcCer when compared to WT. Newborns display a particularly aggressive phenotype, with cyanosis, lethargy and wrinkled reddish skin. Tissue analysis revealed lipid storage cells in liver, spleen, bone marrow and brain [61,62]. Ultrastructural analysis revealed that, in spite of GlcCer presence in the lysosomes of macrophages, microglia and, in low amounts, motor neurons, no alterations were found in overall architecture of liver, bone marrow, spleen and brain [63]. Absence of active GCase during development results in accumulation of GlcCer and absence of free ceramides in the skin´s stratum corneum, originating a previously unrecognized loss of skin barrier phenotype in GD [62,64]. These animals had severe breathing problems and do not survive beyond 24 h. While the model did not reproduce recognizable human GD symptoms, it revealed decreased Bcl-2, BDNF and NGF expression in fetal brains (E19.5), coinciding with an increase in pro-inflammatory factors, reactive oxygen and apoptosis [65,66,67].

A number of strategies we used to generate less severe models that would resemble human GD manifestations. Knock-in strategies for the GD type 2 (RecNcil) and type 3 (L444P) mutations produced homozygous mutants that had around 4–9% of residual GCase activity (RecNcil) and 20% (L444P). Notably, the RecNcil homozygote accumulated GlcCer in liver and brain while the L444P homozygote did not. Nonetheless, pups had severe skin phenotypes and, similarly to the nGBA model, died in the first 2 days of life. These models are now considered to replicate the symptoms of “collodion GD babies”, in which severe GD genotypes present congenital ichthyosis [68]. A two-step breeding strategy combined with optimized husbandry methods can be used to obtain L444P adult homozygotes. These mice had GCase activity levels not surpassing 15–20% activity of the WT (similar to GD type 1 patients); although there was no observable Gaucher cells, the mice showed some GD features such as lymphadenopathy, anemia and generalized inflammation marked by elevation of TNF-α and IL-1β [69].

Mouse models for the point mutations N370S, V394L, D409H and D409V were generated via homologous recombination [70]. In marked contrast to human N370S homozygous patients, N370S homozygote mice died briefly after birth due to a skin phenotype resembling the nGBA homozygotes. All the other homozygote genotypes, V394L, D409H and D409V, survived and were fertile. Despite inter-tissue variation, GCase activity was never higher than 25% of the WT. Scattered Gaucher cells were found after seven months in D409H/D409H and D409V/D409V mice, and after 12 months in V394L/V394L mice. Any of these mutations in heterozygosis with a null allele resulted in more severe phenotypes, presenting moderate levels of GlcCer accumulation, Gaucher cells in lungs and liver three months earlier than in the corresponding homozygotes [70] and memory loss after one year of age [71,72]. However, other GD symptoms were generally absent.

Nonetheless, these models have been used for a number of studies. D409H/D409H and V394L/V394L have been used to test the effect of alterations in calcium homeostasis [73,74,75]. Combined with CBE administration, these same genotypes have been used to study progression of the neuronopathic phenotype [58] and the role of saposin C on autophagy impairment [76]. Expression analysis of GD phenotype progression in V394L/V394L and D409 V/null revealed a direct relationship between GlcCer accumulation and activation of INFγ and IL4 regulated networks [77]. Moreover, these models were used to test a glucosylceramide synthase inhibitor (Eliglustat) which successfully prevented GlcCer accumulation [78] either alone, or in combination with the recombinant enzyme imiglucerase [79]. In addition, intravenous administration of an AAV8 vector expressing human GCase in pre-symptomatic D409V/null mice resulted in prevention of GlcCer accumulation [80].

In order to overcome the neonatal lethality associated with skin defects, conditional KO approaches were used. The first approach was a mouse which contained loxP sites flanking *GBA1* exons 9–11 in combination with a transgene driving CRE recombinase from an inducible promoter. GCase deletion after birth avoids skin development defects due to lack of GCase during embryogenesis. Recombination occurred with high efficiency in spleen, bone marrow and liver and with less efficiency in the brain. Histological analysis revealed presence of Gaucher cells in spleen, bone marrow and liver but not in the brain, skin or kidney [27]. After 12 months GlcCer levels increased ~100 fold in bone marrow and spleen, and ~50 fold in the liver [27]. Bone marrow transplantation, even with low engraftment, is able to ameliorate GD symptoms and eliminate Gaucher cells [27,81]. Deletion of exons 8–11 suing the same strategy produced a more severe phenotype, including osteonecrosis, thymic T-cell aberrations and skeletal defects [82]. This model proved that the major cause for skeletal defects in GD was osteoblastic malfunction and not enhanced bone resorption as previously thought [82]. A third approach aimed to specifically knock out GCase in hematopoietic-endothelial cells by driving CRE recombinase expression from an haematopoietic specific promoter, leading to a generalized decrease in GCase activity and GlcCer accumulation in liver, spleen, leukocytes and brain [83]. However, these models had little or no effect on the neuronal phenotype.

Neuronal mouse models for GD were obtained via two different conditional knock out strategies. The first strategy was to cross mice carrying a loxP flanked Neo insertion in *GBA1* exon 8 (in heterozygosis) to mice expressing CRE recombinase under a epidermis specific promoter (K14), resulting in GCase knock out in al tissues except the epidermis [84]. As expected, the offspring did not show the neonatal skin phenotype and survived; in the course of two weeks, they developed violent and progressive neurological symptoms associated with microglia activation and neuronal loss, motor dysfunction, hyperextension of the neck and finally end-stage paralysis. GlcCer accumulation in brain, spleen and liver, concordant with what is seen in GD type 2 patients [84]. The second strategy involved generation of a CNS specific GCase knock out by way of crossing mice with the complete *GBA1* gene flanked by loxP sites with a mouse driving CRE recombinase from a CNS specific promoter (nestin). The resulting offspring lacked GCase specifically in the neuronal and glial lineages [85] and developed a similar neurological phenotype (albeit ~8 days later).

## 5. Induced Pluripotent Stem Cells for Disease Modeling

While the value of mouse models for modeling GD is undisputed, they still suffer from the disadvantage of not being human. Species-specific differences in genomic regulation, anatomy and physiology can limit the validity of murine results in the human system. In 2006, Takahashi and Yamanaka demonstrated that human somatic cells can be reversed to a pluripotent state by direct reprogramming via ectopic expression of four transcription factors, Oct4, Sox2, Klf4 and c-Myc [86]. Subsequently, a range of methods combining use of different transcription factors, gene delivery methods and small molecules were developed [87]. A direct consequence of this development was the possibility of creating patient specific induced human pluripotent stem cells (iPScs) as in vitro models of human disease. In fact, disease specific iPSc have been successfully derived from patients suffering from a wide range of pathologies [88], including Rett Syndrome [89], Amyotrophic Lateral Sclerosis [90], Spinal Muscular Atrophy [91], Fanconi Anemia [92] and Long Q-T Syndrome [93], among others. Several lysosomal storage diseases have also been modeled in iPSc, including Gaucher’s disease [94].

## 6. Induced Human Pluripotent Stem Cells (iPSc) Modeling of Pathogenic Mechanisms in GD

Several groups have created iPSc models from GD patients with various genotypes and differentiated iPSc to macrophages, neurons or both (Table 2). In 2011 Mazzulli et al. reported the first use of a GD iPSc model for mechanistic studies [95]. Having obtained data using a human neuroglioma cell line (H4) indicating that knockdown of GCase impeded clearance of α-synuclein, these authors used GD iPSc derived neurons to validate their results. Fibroblasts from a N370S/84GG insertion GD patient were reprogrammed via retroviral transduction with Oct4, Sox2, Klf4 and c-Myc. They were shown to express pluripotency markers, and have a normal karyotype and genomic structure. Somewhat unconventionally, their ability to differentiate into all three germ layers in vitro or through teratoma formation was demonstrated in a later publication [96]; however, the iPSc cell line was differentiated to produce cultures that were 80% positive for TUJ1 neurons using a dual inhibition of the SMAD signaling pathway approach with up to 10% dopaminergic neurons (TH positive). As expected, the neurons had low levels of GCase protein and activity as compared to a WT control. Interestingly, these neuronal cultures contained four-fold higher levels of α-synuclein than the control, while other neuronal proteins with a tendency to accumulate (Tau, huntingtin) showed little or no change.

In 2012 Panicker et al. [97] derived and fully characterized iPSc lines for the three clinical types of GD: Type 1 (N370S/N370S), type 2 (L444P/RecNcil) and type 3 (L444P/L444P). Each clone differentiated into both macrophages and neurons. All lines presented the hallmark phenotypes of GD, with sphingolipid accumulation and low levels of GCase. GD-Macrophages took significantly longer to phagocyte red blood cells (RBC), indicating a lysosomal defect. The phagocytic burden increased with the severity of the mutation. Furthermore, macrophages expressed higher levels of tumor necrosis factor α, IL-6 and IL-1bβ, reproducing high levels of inflammatory agents present in serum of GD patients. Moreover, the phagocytic phenotype was reversed with the supplementing the media with recombinant GCase or by treating with either the pharmacological chaperone isofagomine or ambroxol [98], in accordance with what has been observed in animal models and clinical trials. Lysosomal depletion and a block in autophagocitic flux were also observed in GD iPSc derived neuronal populations. Interestingly, these neurons were found to have lower levels of TFEB (transcription factor EB, a key regulator of lysosomal genes) and lysosomal gene expression compared to controls. Overexpression of GCase reversed the autophagic phenotype, but over-expression of TFEB did not; however, overexpression of TFEB did enhance the phenotypic recovery afforded by GCase overexpression [99].

Schöndorf et al. extended iPSc modeling of GD disease by deriving lines from PD patients carrying *GBA1* heterozygous mutations RecNcil, L444P and N370S [100]. The *GBA1* mutations were corrected in three lines using ZFN mediated homologous recombination, creating isogenic sets of lines. IPSCs were differentiated to neuronal populations using a floor plate neural induction protocol that resulted in up to 20% of cells expressing dopaminergic markers (TH, FOXA2, NURR1, GIRK2, and VMAT2), which were further purified by FACS sorting for a combination of CD24high, CD29-, CD184-, CD44- and CD15-, creating a highly-enriched population of dopaminergic neurons. The resulting cells were electro-physiologically normal and differentiation rate and efficiency were not affected by *GBA1* mutations tested. Fully differentiated and FACS purified heterozygous iPS-derived neurons harboring the mutations L444P, RecNcil and N370S were found to recapitulate the sialo-ganglioside profile found in the brain (higher levels of GM1, GD1a, GD1b, GT1b as compared to GD3, GD2 and GQ1b. Neuronal cultures harboring *GBA1* mutations showed lower levels of GCase, increased accumulation of GlcCer and higher levels of α-synuclein. Significantly, these phenotypes were observed both in the *GBA1* mutant homozygous lines vs. WT controls as well as the PD lines carrying heterozygous *GBA1* mutations, with clear differences from the mutation corrected isogenic controls, demonstrating that *GBA1* heterozygotes, while usually devoid of clinical symptoms, do show cellular phenotypes. Furthermore, the effect of both homozygous and heterozygous *GBA1* mutations was shown to extend to an impaired autophagic flux. Finally, proteomic analysis of fully differentiated iPS-derived mDA neurons carrying the L444P allele revealed upregulation of neuronal calcium-binding protein 2 (NECAB2), which correlated with a deficient calcium-buffering response to ER stress; these results suggest *GBA1* mutations may lead to increased calcium mediated neurotoxicity and neurodegeneration [100].

Using neurons differentiated from iPSc lines derived from GD Type 2 patient fibroblasts (L444P/P415R; G325R/C342G; L444P:E326K/L444P:E326K), Sun et al. [101] also found decrease in GCase and increases in upstream substrates. Interestingly, GD type 2 neurons consistently accumulated more glucosylsphingosine than glucosylceramide, the levels of which were dependent on genotype and developmental stage. Accumulation of α-synuclein was suggested by immunofluorescence in neurons of two of the lines and conclusively shown by western blot in only one line. Electrophysiological analysis by patch-clamp revealed reduced negative resting membrane potentials in GD type 2 neurons as compared to WT controls. This finding was also observed in CBE treated WT neurons, indicating the defect to be related to GCase activity levels. Of note, a GD heterozygous iPSc line (L444P:1483G>C:1497G>C) was also developed, but not thoroughly characterized: while GCase levels were about half of WT levels and both glucosylsphingosine and glucosylceramide were unchanged, levels of α-synuclein and electrophysiological activity were not determined; therefore, an independent confirmation of heterozygote phenotypes found by Schöndorf et al. is still needed. These results suggest that further investigation of the connection between neuronal electrophysiological activity with decreased potassium pump gene expression and mitochondrial output are warranted.

More recently, Aflaki et al. [102] derived iPSc lines from GD patients (N370S/N370S; N370S/84GG; IVS2+1G>T/L444P). Of note, the study included two patients who were suffering from PD in addition to GD. Macrophages and dopaminergic neurons derived from these lines also showed decreased GCase activity and increased levels of glucosylceramide and/or glucosphingosine, but were electrophysiologically normal. Contrary to Sun et al. [101], there was no mention of glucosphingosine levels increasing more consistently than those of glucosylceramide, or of reduced resting potentials. Dopaminergic neurons from both GD and GD + PD patients were found to be defective in dopamine re-uptake, but GD + PD were more severely affected.

## 7. Drug Discovery Using iPSc Models

In addition to investigation of mechanisms of pathogenesis, disease specific iPSc can be used for screening and development of pharmacological compounds for therapy. As discussed above, ERT has the disadvantage of being expensive; in addition, it does not impact neurological symptoms due to its inability to cross the blood–brain barrier due to its size. Therefore, screening for smaller compounds or developing them though design is a promising approach, and a number of compounds with various chemistries have been developed [103,104]. Some of these compounds have chaperone activity and increase levels of active GCase by enhancing correct folding during trafficking to the lysosome, but others may act though alternative mechanisms. Furthermore, the inverse relationship between GCase activity and α-synuclein accumulation first proposed by Mazzulli et al. [95] suggested that any approach enhancing GCase could benefit PD patients as well as GD patients. Using an iPSc model, our group has tested two bicyclic sp2-iminosugars (nojirimycin analogs) in dopaminergic neurons derived from a GD type 2 iPSc line (L444P/G202R) with promising results [105]. These compounds are substrate mimetics that interact reversibly with the active site of GCase and could stabilize the enzyme enough to help it escape the ER quality control system; protein and activity levels increased up to four-fold.

While chaperone candidates can be identified through in vitro cell free assays or in GD fibroblasts, the iPSc model allows testing of the effect of candidate chaperones on neuron specific phenotypes such as electrophysiology and α-synuclein accumulation or clearance. Recently Aflaki et al. [102] reported a salicylic acid derivative (NCGC607) with beneficial effects on several cellular phenotypes of iPSc derived dopaminergic neurons, including increased GCase levels, decreased substrate levels and decrease in accumulation of α-synuclein. This finding has raised the possibility that modulators of GCase activity could reverse α-synuclein accumulation both in GD and PD patients. In addition, a second GCase modulator, NCGC00188758 [106], effectively increased α-synuclein clearance in dopaminergic neurons differentiated from iPSc derived from PD patients; notably, the patients studied included both idiopathic PD and PD caused by mutations in α-synuclein (*SNCA*) (triplication or A53T) or ATPase13A2 (*PARK9*) genes. In addition, the beneficial effect was also found in GD Type 1 patients (N370S/c0.84dupG), GD type 2 patients (IVS2+1G>T/L444P) and a patient with GD type 1 genotype N370S/N370S who also suffered from PD. A summary of these studies is presented in Table 3.

## 8. Conclusions

Disease modeling with iPSc has several distinct advantages. First, iPSc lines have virtually no expansion limit and therefore provide an inexhaustible source of cells for study. Second, the mutation causing the disease is naturally present in the original cells reprogrammed; this precludes the need for complex procedures to create the genetic defect and avoids potential secondary effects of the genetic engineering approach chosen. Third, under the right conditions, iPSc can differentiate into virtually any cell type in vitro, providing a source of disease relevant cells. In the case of GD, a number of protocols have been developed that allow differentiation to monocytes/macrophages and several subtypes of neurons [95,98,105]. Finally, the cells are of human origin and species specific differences are avoided. The combination of these four advantages makes iPSc disease modeling a powerful preclinical model for study of basic pathogenic mechanisms and development of potential therapeutic, and an important complement to animal model systems [88]. In recent years, the GD experimental paradigm has shifted somewhat from mouse models to iPSc models. This shift has allowed new insights into a number of aspects of GD molecular and cellular pathology, including, as described throughout this review, electrophysiology, calcium signaling, inflammatory response, autophagic flux, lysosomal defects and, importantly, α-synuclein accumulation and the mechanistic relationship between GD and the Parkinson’s related synucleinopathies. In particular, iPSc modeling may prove useful to finally answer the question of how much of the GD phenotype is cause by loss of function vs. gain of function mechanisms.

## Figures and Tables

**Table 1:** Comparison of Type 1, Type 2 and Type 3 Gaucher’s disease.

Disease Characteristics	Type 1	Type 2	Type 3
Age of onset	Variable, anywhere between adolescence and old age	Neonatal/infancy	Infancy/early childhood
Frequency	General population: 1 in 100,000; Ashkenazi Jewish population: 1 in 450	1% of GD cases	General population: 5% of GD cases; Swedish Norrbottian Population: 1 in 50,000
Visceral symptoms	Hepatomegaly, splenomegaly, interstitial lung disease, anemia, thrombocytopenia, bone disease	Hepatomegaly, Splenomegaly, Interstitial lung disease, Anemia, Thrombocytopenia	Hepatomegaly, Splenomegaly, Interstitial lung disease, Anemia, Thrombocytopenia, Bone disease
Neurologic symptoms	None (except those derived from possible development of Parkinson-like synucleinopathy)	Acute neurologic problems, spasticity, seizures, convulsions, severe neuro-degeneration	Chronic neurologic problems, seizures, eye movement disorders, poor coordination, cognitive problems
Severity	Asymptomatic to severe	Death between 1 and 3 years of age	Severe before adulthood.

**Table 2:** Summary of GD induced human pluripotent stem cells (iPSc) models in terms of genotypes, clinical presentation, reprograming method, cell types differentiated to, main findings and citations.

Genotype	GD Type	Derivation Method	Differentiated to	Main Contribution/Findings	Citation
N370S/84GG	GD1	Retrovirus	Neurons	Proposal of a bidirectional GCase-α-synuclein forward feed mechanistic loop.	[95]
N370S/N370S L444P/RecNciI L444P/L444P	GD1 GD2 GD3	Lentivirus	Macrophages Neurons	Establishment of GD iPSc-derived in vitro model; Establishment of erythrocyte clearance as a functional assay for GD macrophages; Functional response to ERT and isofagomine.	[97]
L444P/G202R	GD2	Lenti LoxP	Macrophages Neurons	Establishment of GD iPS-derived in vitro model; Chaperone compounds for increased GCase activity in neurons.	[105]
L444P/WT RecNciI/WT N370S/WT N370S/N370S L444P/L444P	WT WT WT GD1 GD3	Retrovirus	Neurons	Establishment of GD iPSc-derived in vitro model; Isogenic lines created via Zn finger mediated recombination; Observation of autophagic defects, NECAB2 overexpression and calcium homeostasis dysregulation	[100]
N370S/N370S L444P/RecNciI W184R/D409H L444P/L444P	GD1 GD2 GD2 GD3	Lentivirus	Macrophages	GD derived macrophages express high levels of the inflammatory mediator’s TNF-α, IL-6, and IL-1β	[98]
N370S/N370S N370S/84GG IVS2+1G>A/L444P	GD1 GD1 GD2	Lentivirus	Macrophages	In vitro characterization of NCGC607 as a small-molecule non-inhibitory pharmacological chaperone for treatment of GD type 2.	[106]
L444P/1483G>C; 1497G>C L444P/P415R G325R/C342G L444P; E326K/L444P; E326K	WT GD2 GD2 GD2	Episome Lentivirus Episome Episome	Neurons	Altered negative resting membrane potential lead to abnormal electrophysiological properties in GD2 iPSc-derived neurons.	[101]
N370S/N370S L444P/RecNciI W184R/D409H L444P/L444P	GD1 GD2 GD2 GD3	Lentivirus	Neurons	Autophagic defects related with *GBA1* mutations are caused by downregulation of autophagy master regulator TFEB.	[99]
N370S/WT	WT	Retrovirus	Neurons	*GBA1* associated PD phenotype is preceded by ER stress, autophagic/lysosomal perturbations, and elevated extracellular α-synuclein in heterozygosity.	[107]
N370S/84GG	GD1	Retrovirus	Neurons	Increase in GCase activity clears α-synuclein and normalizes autophagy in PD patient neurons; α-synuclein disrupts the lysosomal flux by affecting protein trafficking in PD.	[96]
N370S/N370S N370S/84GG IVS2+1G>T/L444P	GD1 GD1 GD2	Lentivirus	Macrophages Neurons	A new GCase chaperone, NCGC607, reduces α-synuclein and glycolipid accumulation in GD and PD patient iPSc-derived neurons.	[102]

**Table 3:** Summary of GD iPSc models in terms of genotypes, cell types differentiated to, chaperone compounds tested, fold increase after chaperon treatment measured, length of chaperone treatment and citations.

Genotype	Measured on	Activity	Chaperone	Activity Increase	Treatment Duration	Reference
N370S/84GG	Neurons	~10%	NA	NA	NA	[95,96,108]
N370S/N370S L444P/RecNciI W184R/D409H L444P/L444P	Macrophages Neurons	~5%	Isofagomine	1,7-2 fold	5 days	[97]
L444P/G202R	Neurons	~15%	NOI-NJ	4-6 fold	4 days	[105]
			6S-ADBI-NJ			
N370S/N370S L444P/RecNciI W184R/D409H L444P/L444P	Macrophages	~5%	Ambroxol	2 fold	3–6 days	[98]
N370S/N370S N370S/84GG IVS2+1G>A/L444P	Macrophages	~21.2% ~17.6% ~2.9%	NCGC00188758	3.2 fold 3.2 fold 26.6 fold	6 days	[106]
L444P/WT RecNciI/WT N370S/WT N370S/N370S L444P/L444P	Neurons	~57% ~57% ~71% ~43% ~28%	NA	NA	NA	[100]
L444P;1483G>C;1497G>C/WT L444P/P415R G325R/C342G L444P; E326K/L444P; E326K	Neurons	~28% ~5,2% ~21,5% ~4,8%	NA	NA	NA	[101]
N370S/N370S N370S/84GG IVS2+1G>A/L444P	Neurons	~40% ~40% ~2%	NCGC607	2 fold 1.8 fold 40 fold	21 days	[102]

## Abbreviations

CBE	Conduritol-β-epoxide
CNS	Central nervous system
ER	Endoplasmic reticulum
ERAD	ER Associated Degradation
ERT	Enzyme replacement therapy
FOXA2	Forkhead class A2 protein
*GBA1*	Acid β-glucocerebrosidase gene *
GCase	Acid β-Glucocerebrosidase enzyme
GlcCer	Glucosylceramide
GD	Gaucher’s disease
GD1a	Ganglioside 1a
GD1b	Ganglioside 1b
GD2	Ganglioside 2
GD3	Ganglioside 3
GIRK2	G protein-activated inward rectifier potassium channel 2
iPSc	Induced pluripotent stem cells
KO	Knockout
LSD	Lysosomal storage disease
mDA	Midbrain dopaminergic
NECAB2	Neuronal calcium-binding protein 2
nGBA	*GBA1* null
NURR1	Nuclear receptor related 1 protein
*PARK9*	Coding gene for ATPase13A2
PD	Parkinson’s disease
*SNCA*	α-Synuclein gene
SRT	Substrate reduction therapy
TFEB	Transcription factor EB
TH	Tyrosine hydroxylase
TUJ1	Class III β-tubulin
UPS	Ubiquitin–proteasome
VMAT2	Vesicular monoamine transporter 2
WT	Wild type
ZFN	Zinc-finger nuclease
* Note: Throughout this work, the traditional nomenclature “*GBA1*” is used instead of the HGVS recommended nomenclature.	
